# Seasonal variation in the soil fungal community structure of *Larix gmelinii* forests in Northeast China

**DOI:** 10.3389/fmicb.2023.1106888

**Published:** 2023-03-22

**Authors:** Wen Zhao, Dan-Dan Wang, Kai-Chuan Huang, Shun Liu, Mumin Reyila, Yi-Fei Sun, Jun-Ning Li, Bao-Kai Cui

**Affiliations:** School of Ecology and Nature Conservation, Beijing Forestry University, Beijing, China

**Keywords:** soil fungi, *Larix gmelinii*, community structure, temporal scale, soil properties

## Abstract

Soil fungi play an indispensable role in forest ecosystems by participating in energy flow, material circulation, and assisting plant growth and development. *Larix gmelinii* is the dominant tree species in the greater Khingan Mountains, which is the only cold temperate coniferous forest in China. Understanding the variations in underground fungi will help us master the situation of *L. gmelinii* above ground. We collected soil samples from three seasons and analyzed the differences in soil fungal community structure using high-throughput sequencing technology to study the seasonal changes in soil fungal community structure in *L. gmelinii* forests. We found that the Shannon and Chao1 diversity in autumn was significantly lower than in spring and summer. The community composition and functional guild varied significantly between seasons. Furthermore, we showed that ectomycorrhizal fungi dominated the functional guilds. The relative abundance of ectomycorrhizal fungi increased dramatically from summer to autumn and was significantly negatively correlated with temperature and precipitation. Temperature and precipitation positively affect the alpha diversity of fungi significantly. In addition, pH was negatively correlated with the Chao1 diversity. Temperature and precipitation significantly affected several dominant genera and functional guilds. Among the soil physicochemical properties, several dominant genera were affected by pH, and the remaining individual genera and functional guilds were significantly correlated with total nitrogen, available phosphorus, soil organic carbon, or cation exchange capacity. For the composition of total fungal community, temperature and precipitation, as well as soil physicochemical properties except AP, significantly drove the variation in community composition.

## 1. Introduction

Soil fungi are important components of soil microorganisms and play a critical role in the energy flow and material cycle of forest ecosystems (Falkowski et al., 2008; Cho et al., 2017). There are some important functional groups of the soil fungi: saprophytic fungi can produce enzymes to decompose organic matter (Voříšková and Baldrian, 2013) promoting the carbon (Rousk and Frey, 2015) and nitrogen cycles (Morrison et al., 2016); mycorrhizal fungi are symbiotic with plants and assist in plant growth and development (Eldridge et al., 2021); some pathogenic fungi can also cause fungal infection of the host and affect its productivity (Maron et al., 2011). While soil fungi can affect plant community structure directly or indirectly, plant litter and root exudates are the major sources of fungal nutrition (Broeckling et al., 2008; Nakayama et al., 2019; Hannula et al., 2021).

The diversity and composition of soil fungal communities show great spatial and temporal variability (Voříšková et al., 2014; Zhou et al., 2016a; Averill et al., 2019). Litter decomposition and phytosynthate allocation represent important factors contributing to the seasonal variation of fungal communities, while environmental conditions are important factors contributing to the geographic variation of fungal communities. The soil fungal community is sensitive to changes in the surrounding environment, and its diversity and activity are regulated by some biotic and abiotic factors such as climate factors (Hawkes et al., 2011; Tedersoo et al., 2014), soil properties (Rousk et al., 2010) and vegetation (Kivlin and Hawkes, 2016; Li et al., 2020). Therefore, observing the changes in soil fungal communities can help us understand the soil health of the ecosystem (Shi et al., 2019).

*Larix gmelinii* is a deciduous tree of Pinaceae with rich timber accumulation and is one of the important timber trees in Northeast China (Sun et al., 2007), which is located in a sensitive area of global warming (Li et al., 2006). As an ectomycorrhizal-associated tree species (Guo, 2012), the ectomycorrhizal fungi of *L. gmelinii* included *Cortinarius*, *Hygrophorus*, *Lactarius*, and *Suillus* (Liu et al., 2014). To date, most of the studies on the soil fungal community in *L. gmelinii* forest have focused on different forest types (Yang et al., 2017), fires (Yang T. et al., 2020), and rhizospheres (Yang Y. et al., 2020), while ignoring the understanding of the fungal community in seasonal changes on a temporal scale.

In this study, we collected 45 soil samples of *L. gmelinii* in three seasons aimed to characterize the soil fungal community in the *L. gmelinii* forest, including taxonomic composition, functional guilds, community structure and to assess the roles of environmental drivers. We hypothesized that, (i) temperature and precipitation will have significant effects on soil fungal communities, which will directly affect the living conditions and activity of fungi, (ii) considering that soil physicochemical properties vary in different seasons in the same location, pH should have an more important role on community structure compared to soil nutrient conditions.

## 2. Materials and methods

### 2.1. Study site

**Figure 1:**
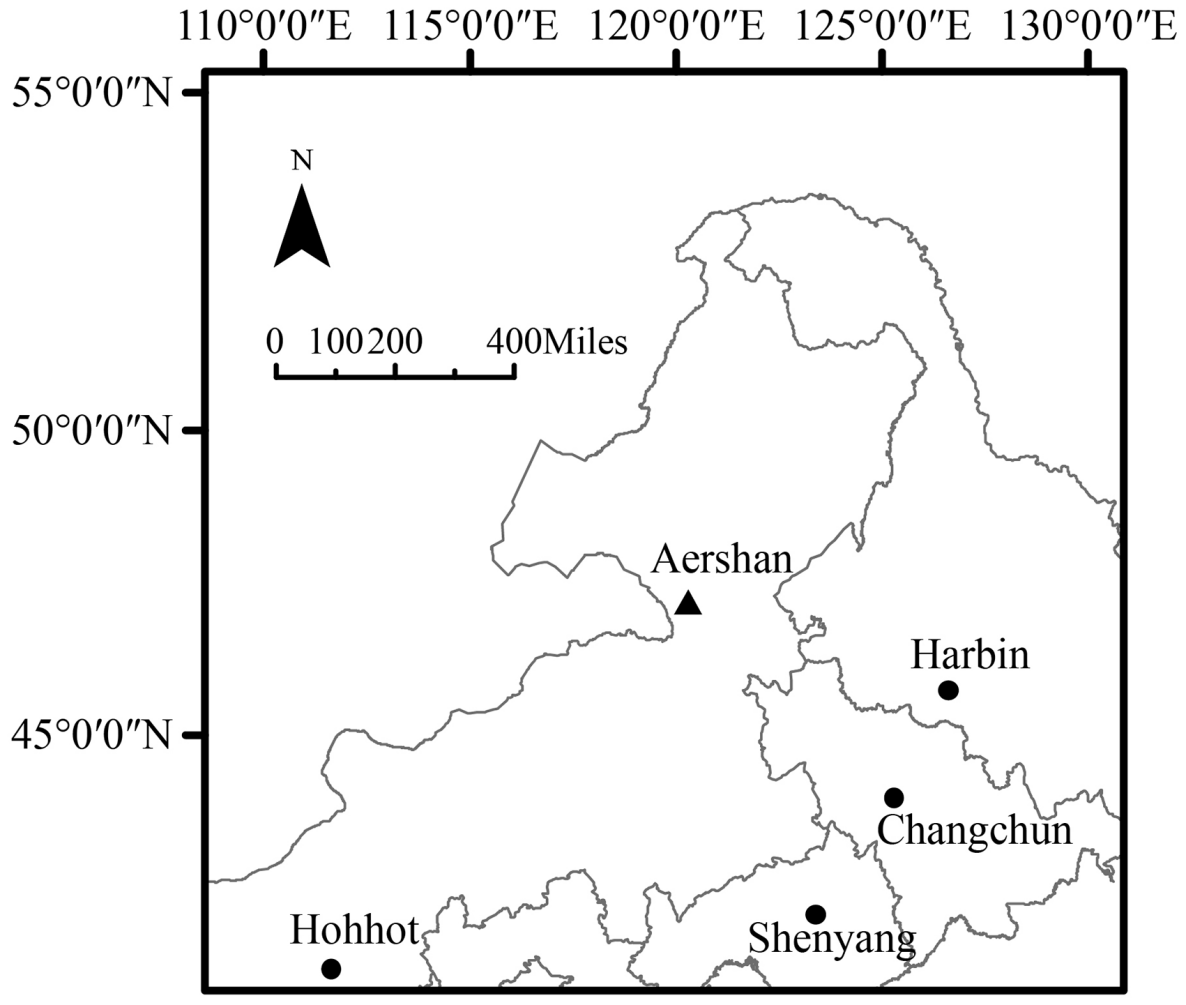
Sampling location in Aershan National Forest Park.

The study area is located in Aershan National Forest Park (47°15′17′N, 120°17′34′E, Figure 1), which is located in Aershan city, Inner Mongolia Autonomous Region, China. Aershan National Forest Park is located in the continental climate region of the Mongolian Plateau and belongs to the cold temperate humid zone. The annual average temperature is −3.2°C, the average precipitation is 452.1 mm (Li et al., 2011). The winter is long and cold, the summer is short and rainy, and the spring and autumn are cool with less precipitation (Liu et al., 2020). The plant growth period is generally 100–120 days. The dominant tree species in the forest area are *L. gmelinii* and *Betula platyphylla*, with a forest coverage rate of 80%. The study area is located in the high latitude permafrost distribution area in China (Ding et al., 2019), and the zonal soil is retisols (IUSS Working Group WRB, 2015).

The *L. gmelinii* artificial forest lies at 1055 m elevation, and the slope of the study area is 12 degrees. The tree density of the forest is 2,500 stems per ha. The tree is nearly 14 m high with a stand age of 40 years. Diameter at breast height (DBH) of the selected trees ranged from 14 to 24 cm, and the average DBH of the trees is 18 cm. The main understory is various types of grass such as *Potentilla freyniana* and *Calamagrostis angustifolia*.

### 2.2. Soil sampling

In May, July, and October 2021, representing spring, summer and autumn respectively, soil samples were collected. Defined May to October as the growing season of *L. gmelinii*. We established three 20 m × 20 m plots at least 100 m apart, and took five soil samples from four vertices and the center as independent samples, for a total of 15 independent samples per season to represent the soil fungi in this stand. A total of 45 samples were taken for this experiment (5 independent samples × 3 plots× 3 seasons). Soil from 0–20 cm were obtained, passed through a 2 mm sieve and mixed, then filled three centrifuge tubes for DNA extraction and a ziplock bag for physicochemical property determination. The samples in centrifuge tubes were temporarily frozen in an incubator filled with dry ice and then transported to the laboratory stored at −80°C for subsequent DNA extraction, and the samples in ziplock bags were dried for determination of soil physicochemical properties. We extracted soil DNA from three separate centrifuge tubes and then mixed them into one to represent the sample.

### 2.3. Environmental variables

For the determination of soil properties, we referred to the method of Bao (2000). The soil in the sealed bag was dried naturally to determine its physicochemical properties: soil organic carbon (SOC) was determined by the potassium dichromate volumetric method, available phosphorus (AP) was determined by molybdenum antimony resistance colorimetry, pH was determined by the potential method, total nitrogen (TN) was determined by Kjeldahl method, the cation exchange capacity (CEC) was determined by the ammonium acetate exchange method (Liang et al., 2015).

Climate data for each season were obtained from WorldClim^1^ (accessed May 19, 2022), including monthly total precipitation (Prec), and monthly maximum and minimum temperature (Tmax and Tmin) from 2010 to 2018. Therefore, the climatic variables (Tmax, Tmin and Prec) are only three levels throughout the samples according to three seasons.

### 2.4. DNA extraction, amplification, and sequencing

A DNeasy Power Soil Pro Kit kit (Qiagen, Frankfurt, Germany) was used to extract the total genomic DNA of the soil samples. A NanoDrop NC2000 spectrophotometer (Thermo Fisher Scientific, Waltham, MA, United States) was used to quantify DNA; the quality of DNA was detected by 1.2% agarose gel electrophoresis. The ITS1 region of fungi was amplified, and the primers were ITS5F (5-GGAAGTAAAGTAAAAGTCGTAAAGG-3) and ITS2R (5-GCTGCGTTCTTCATCGATGC-3) (Bellemain et al., 2010), PCR system (20 μL): 2 μL (2.5 mM) dNTP. 1 μL (10 μM) forward primers and 1 μL (10 μM) reverse primers, 2 μL template DNA, 10 μL ddH2O, and 4 μL Fast pfu DNA polymerases. Circulating system: 95°C for 2 min; 30 cycles at 95°C for 30 s, 55°C for 30 s, and 72°C for 30 s; 72°C for 5 min. The PCR amplification was performed by Applied Biosystems 2,720 Thermal Cycler (Thermo Fisher Scientific, Waltham, MA, United States); the Quant-iT PicoGreen dsDNA assay was performed by a Microplate Reader FLx800 (BioTek, Burlington, Vermont, United States). PCR amplicons were purified and recovered by adding Vazyme VAHTSTM DNA Clean Beads (Vazyme, Nanjing, China) and quantified with the fluorescent reagent of Quant-iT PicoGreen dsDNA assay kit (Invitrogen, Carlsbad, CA, United States); then, amplicons were mixed in proportion to the sequencing amount, and pair-end 2 × 250 bp sequencing was performed using the Illlumina MiSeq platform with a NovaSeq 6000 SP reagent kit at Shanghai Personal Biotechnology Co., Ltd. (Shanghai, China). All raw sequencing data of this study are deposited into the NCBI database with the Short Read Archive (SRA) accession number PRJNA903950.

### 2.5. Sequence analysis

The early sequence processing of this experiment was based on QIIME 2–2021.2 (Bolyen et al., 2019). The Demux plug-in was used to split samples, the DADA2 plug-in was used to perform quality control, such as filtering and noise removal, position at which forward and reverse read sequences should be truncated due to decrease in quality were 222 and 232 respectively, the min and max sequence length were 230 and 438, forward and reverse reads with number of expected errors higher than 2 were be discarded. The Vsearch plug-in was used to cluster sequences into operational taxonomic units (OTUs) according to the 97% similarity principle, and the phylogeny plug-in was used to control and generate phylogenetic trees. Then, according to the fungus UNITE v.8.2 database (Nilsson et al., 2019), we used the feature classifier plug-in to annotate the species. The function guilds and trophic modes were obtained from the FUNGuild database (Nguyen et al., 2016). Finally, we used the rarefy command of the vegan package in R (R Core Team, 2022) to standardize the OTU table according to the minimum sequence number of samples, and made the rarefaction curve by rare curve command.

### 2.6. Statistical analysis

The data processing part was mainly completed with R 4.1.2 and SPSS 26.0 (SPSS Inc., Chicago, IL, United States), and the R part was mainly processed with the vegan package (Oksanen et al., 2020). In alpha diversity analysis, the Shannon diversity index, Chao1 richness index, and Pielou evenness index were selected for one-way analysis of variance (ANOVA) and *t*-test to compare species diversity in different seasons. To identify species and guilds enriched in seasons we employed linear statistics on relative abundance values using the R package edgeR. We grouped taxa and functions that were not identified or whose average relative abundance was less than 1% into “others,” and defined dominant taxa as which relative abundance more than 1%. The beta diversity was tested by non-metric multidimensional scaling (NMDS) based on the OTU level, and the difference between the three fungal communities was tested by analysis of similarities (ANOSIM) of 999 permutations. Redundancy analysis (RDA) was used to explore the relationship between fungal community structure and soil physicochemical properties. The envfit function was used to test the significance of each physicochemical factor in RDA, and the Mantel test was conducted on community structure and soil physicochemical properties. Spearman correlation was used in Mantel test, and 999 random permutations were set to obtain the correlation R-value and the significance *p*. SPSS 26.0 was used to analyze the differences between groups of soil physicochemical properties using ANOVA, and the Spearman correlation coefficient was used to analyze the correlation between alpha diversity, dominant genera, functional guilds of fungal communities and soil physicochemical properties.

## 3. Results

### 3.1. Soil properties and climate factors

The soil total nitrogen (TN), available phosphorus (AP), and cation exchange capacity (CEC) were not significantly different among the three seasons (Table 1). Soil organic carbon (SOC) showed a significant upwards trend with seasons from spring to winter (*p* < 0.05). pH decreased in summer and increased in autumn (*p* < 0.001). Temperature and precipitation gradient showed a strong rise in summer and then decline in autumn, and the temperature and precipitation in autumn were much lower than those in spring.

**Table 1:** Soil properties and climate factors compared by ANOVA (mean values ± S.E.s).

Factor	Season	Spr.	Sum.	Aut.	*F*-value	*p*
Soil property	TN/(g/kg)	5.638 ± 0.135a	5.786 ± 0.124a	5.769 ± 0.165a	0.323	0.726
	SOC/(g/kg)	67.697 ± 1.562b	72.211 ± 1.177ab	75.560 ± 2.482a	4.678	0.015
	AP/(mg/kg)	9.206 ± 0.500a	10.591 ± 0.871a	10.045 ± 0.827a	0.863	0.429
	pH	5.612 ± 0.026b	5.022 ± 0.052c	5.815 ± 0.049a	88.849	0.000
	CEC/(cmol(+)/kg)	47.471 ± 0.435a	46.715 ± 0.385a	46.002 ± 0.572a	2.435	0.100
Climate factor	Tmax (°C)	15.730 ± 0.590b	23.130 ± 0.419a	6.415 ± 0.676c	214.579	<0.001
	Tmin (°C)	1.059 ± 0.430b	10.831 ± 0.215a	−7.928 ± 0.412c	659.990	<0.001
	Prec (mm)	47.204 ± 7.136b	140.721.646a	17.280 ± 2.607b	23.627	<0.001

SOC, soil organic carbon; AP, available phosphorus; pH, pH value; CEC, cation exchange capacity; Tmax, the monthly minimum temperature; Tmin, the monthly minimum temperature; Prec: mean monthly precipitation. Different letters indicate significant differences among different season tested by one-way ANOVA (*p* < 0.05). Soil property data were obtained from 15 replicates, and climate data were obtained from 9 years (2010–2018).

### 3.2. Diversity of the soil fungal community

**Figure 2:**
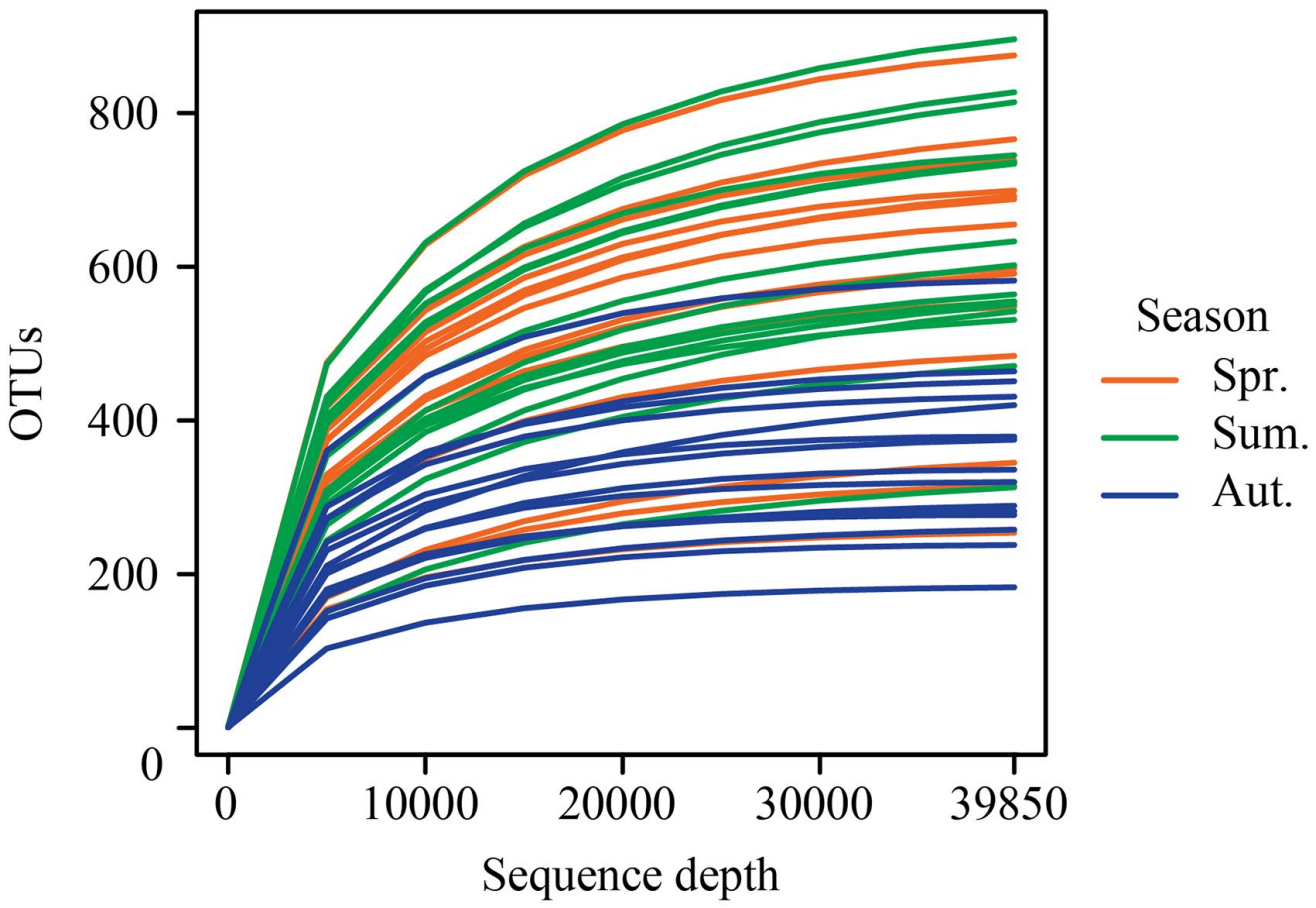
The rarefaction curves of the sequence depth and the number of OTUs from 45 samples.

A total of 2,963,468 quality-filtered sequences were retrieved from the 45 samples. After rarefying 39,850 sequences per sample, a total of 1,793,250 unique sequences were clustered into 5,681 operational taxonomic units (OTUs) at 97% identity. Rarefaction curves (Figure 2) of the number of OTUs with increasing sequence depth of samples, indicating that the rarefied sequence depth in our study capture most fungi members from each season.

**Figure 3:**
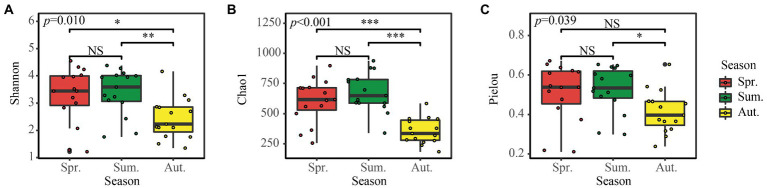
**(A–C)** Alpha diversity of the soil fungal community in the three seasons. NS, Not Significant; *p < 0.05; **p < 0.01; ***p < 0.001.

The Shannon diversity index, Chao1 richness index, and Pielou evenness index, which represent fungal diversity (Figure 3), showed no significant difference between spring and summer, but the Shannon and Chao1 diversity in autumn was significantly lower than in spring and summer.

### 3.3. Community composition of the fungi

**Figure 4:**
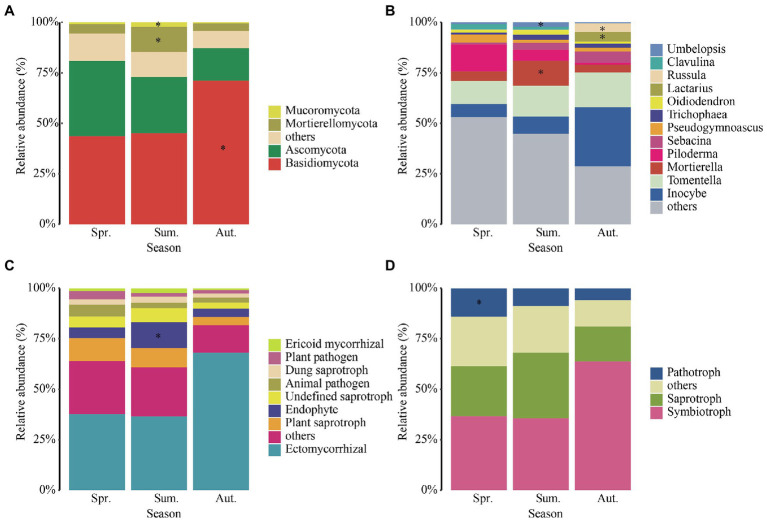
Relative abundances of dominant fungal phyla **(A)**, genera **(B)**, functional guilds **(C)**, and trophic modes **(D)** in each season. The phyla, genera, functional guilds, and trophic modes with less than 0.01of the average relative abundance are grouped into “others.” Asterisks indicate significant enrichment (FDR, *p* < 0.05).

The OTUs belong to 13 phyla, 46 classes, 107 orders, 248 families, 559 genera, and 773 species after classified. There were four dominant phyla with relative abundances more than 1%: Basidiomycota was the most abundant phylum (Figure 4A), followed by Ascomycota, then Mortierellomycota and Mucoromycota. The Mortierellomycota and Mucoromycota were enriched in summer and Basidiomycota was enriched in autumn.

The dominant genera (Figure 4B) which relative abundances more than 1% of fungi were *Inocybe*, *Tomentella*, *Mortierella*, *Piloderma*, *Sebacina*, *Pseudogymnoascus*, *Trichophaea*, *Oidiodendron*, *Lactarius*, *Russula*, *Clavulina*, and *Umbelopsis*. We found that the relative abundance of *Inocybe* increased significantly in autumn, *Pilodermas* decreased significantly with seasonal changes, and the relative abundance of *Mortierella* was higher in summer. In addition, the relative abundance of *Lactarius* and *Russula* increased significantly in autumn. Mortierella and Umbelopsis were significantly enriched in summer. Moreover, Russula and Lactarius were significantly enriched in autumn.

We annotated the taxa and found that the relative abundance of ectomycorrhizal fungi (Figure 4C) was the highest among the annotated functions, followed by plant saprotroph, endophyte, undefined saprotroph, animal pathogen, dung saprotroph, plan pathogen and ericoid mycorrhizal fungi. In addition, the relative abundance of ectomycorrhizal fungi increased dramatically from summer to autumn, up to 66%. The endophyte was significantly enriched in summer. For the trophic mode (Figure 4D), the relative abundance of symbiotroph was significantly high in autumn and the relative abundance of saprotroph was highest in summer. The relative abundance of pathogenic fungi decreased with the growing season and enriched in spring.

**Figure 5:**
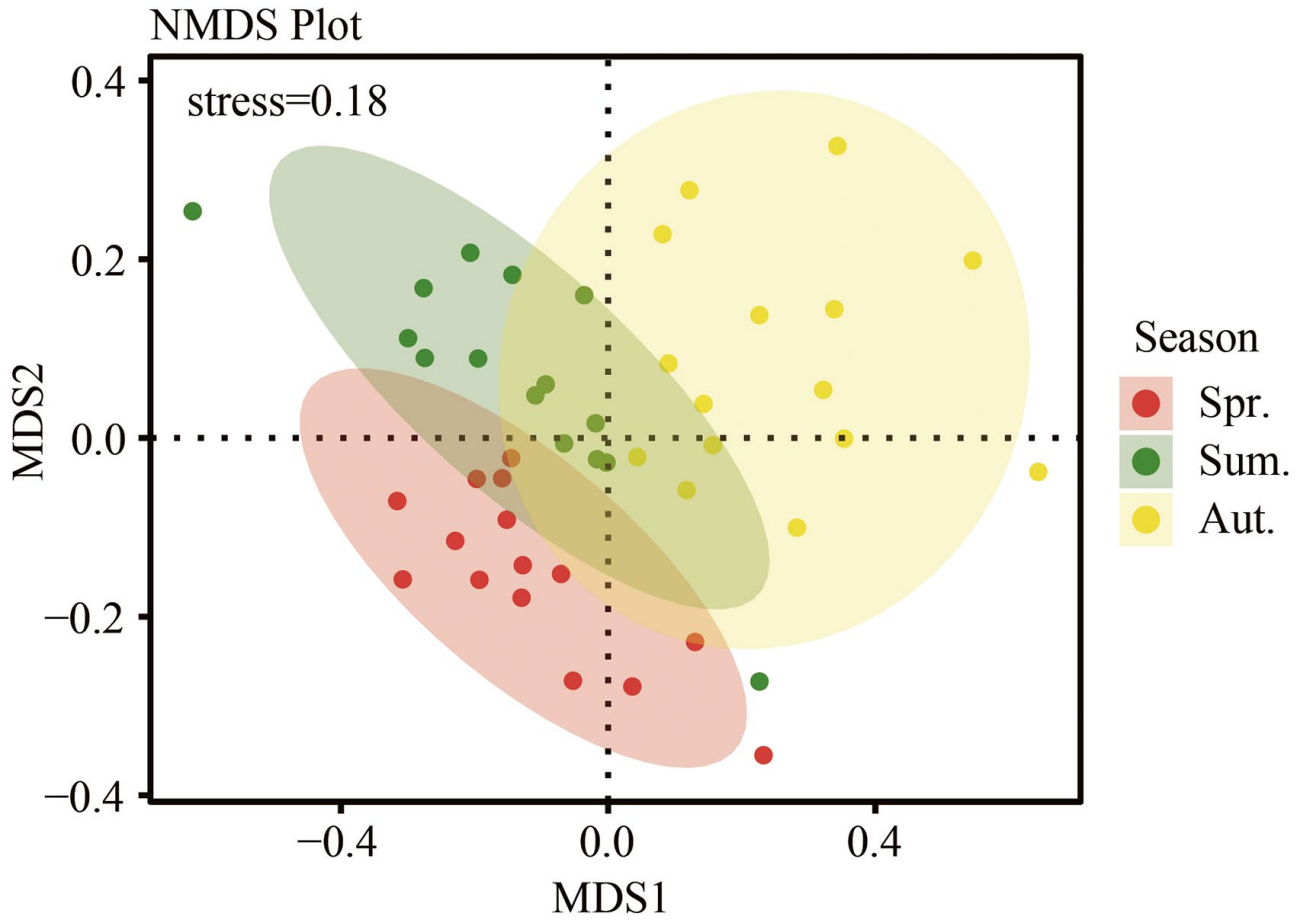
Fungal community structure based on Bray-Curtis distance, as determined by nonmetric multidimensional scaling (NMDS).

The soil fungal community structure in different season was analyzed using nonmetric multidimensional scaling (NMDS) based on the Bray-Curtis distance. We showed that the results of NMDS analysis had some explanatory significance (stress = 0.18). There was some overlap among the three seasons (Figure 5), and there were also unique parts of each, showing the similarities and differences in the community structure of the three seasons. Analysis of similarities (ANOSIM) agreed with the NMDS in that seasonal variation caused differences in fungal community structure (*R* = 0.140, *p* = 0.001).

### 3.4. Environmental drivers of fungal diversity

The Shannon diversity, Chao1 richness and Pielou evenness indices of fungi significantly positively correlated with Tmax, Tmin, and Prec (*p* < 0.05, Table 2). pH was significantly negatively correlated with the Chao1 index of fungal richness (*p* < 0.001). Compared with soil physicochemical properties, climate factors had a stronger impact on fungal diversity.

**Table 2:** Spearman correlation coefficient of alpha diversity to soil properties.

Alpha diversity	TN	SOC	AP	pH	CEC	Tmax	Tmin	Prec
Shannon	0.085	−0.142	−0.176	−0.278	0.114	0.404**	0.404**	0.404**
Chao1	0.185	−0.057	−0.036	−0.532***	0.219	0.652***	0.652***	0.652***
Pielou	0.052	−0.166	−0.205	−0.217	0.081	0.348*	0.348*	0.348**

SOC, soil organic carbon; AP, available phosphorus; pH, pH value; CEC, cation exchange capacity; Tmax, the monthly minimum temperature; Tmin, the monthly minimum temperature; Prec, mean monthly precipitation. *p < 0.05; **p < 0.01; ***p < 0.001.

### 3.5. Environmental drivers of dominant fungal genera and guilds

We conducted Spearman correlation analysis on the dominant genera of fungi and environmental factors (Table 3). We found that *Inocybe* was significantly positively correlated with pH (*p* < 0.001) and negatively correlated with climate factors (*p* < 0.01). In contrast, *Mortierella*, *Piloderma* and *Umbelopsis* were significantly negatively correlated with pH (*p* < 0.01) and positively correlated with climate factors (*p* < 0.05). *Pseudogymnoascus* was negatively correlated with AP (*p* < 0.05). *Oidiodendron* was significantly negatively correlated with SOC (*p* < 0.05). *Russula* (*p* < 0.01) was significantly negatively correlated with CEC. *Clavulina* was influenced by many factors including SOC, pH, CEC and climate factors (*p* < 0.05).

**Table 3:** Spearman correlation coefficients of dominant fungal genera and functional guilds to soil properties.

Factor	Variable	TN	SOC	AP	pH	CEC	Tmax	Tmin	Prec
Genus	*Inocybe*	−0.106	0.060	−0.187	0.464***	−0.197	−0.436**	−0.436**	−0.436**
	*Tomentella*	0.050	0.112	0.045	−0.041	−0.224	−0.040	−0.04	−0.04
	*Mortierella*	0.132	−0.002	−0.126	−0.492***	0.048	0.597***	0.597***	0.597***
	*Piloderma*	−0.023	−0.145	−0.012	−0.389**	0.027	0.361*	0.361*	0.361*
	*Sebacina*	0.050	0.037	−0.027	−0.215	0.280	0.151	0.151	0.151
	*Pseudogymnoascus*	0.093	−0.125	−0.297*	0.200	0.131	−0.101	−0.101	−0.101
	*Trichophaea*	0.077	0.093	0.041	0.069	−0.258	0.016	0.016	0.016
	*Oidiodendron*	−0.220	−0.335*	0.032	−0.061	0.075	0.285	0.285	0.285
	*Lactarius*	−0.030	0.012	−0.167	0.172	0.060	−0.251	−0.251	−0.251
	*Russula*	−0.085	0.058	−0.04	0.134	−0.422**	−0.191	−0.191	−0.191
	*Clavulina*	−0.211	−0.317*	0.077	−0.390**	0.395**	0.393**	0.393**	0.393**
	*Umbelopsis*	−0.138	−0.245	0.033	−0.498***	0.098	0.634***	0.634***	0.634***
Guild	Ectomycorrhizal	0.033	0.218	0.116	0.257	−0.097	−0.474***	−0.474***	−0.474***
	Plant saprotroph	−0.115	−0.142	0.037	−0.009	0.289	0.089	0.089	0.089
	Endophyte	0.124	−0.026	−0.143	−0.471***	0.045	0.591***	0.591***	0.591***
	Undefined saprotroph	−0.009	−0.189	−0.135	−0.383**	0.225	0.497***	0.497***	0.497***
	Animal pathogen	0.138	−0.117	−0.229	0.111	0.193	0.006	0.006	0.006
	Dung saprotroph	0.127	−0.017	−0.091	0.021	−0.142	0.094	0.094	0.094
	Plant pathogen	−0.077	−0.315*	−0.145	−0.037	0.198	0.107	0.107	0.107
	Ericoid mycorrhizal	−0.22*	−0.335	0.032	−0.061	0.075	0.285	0.285	0.285

SOC, soil organic carbon; AP, available phosphorus; pH, pH value; CEC, cation exchange capacity; Tmax, the monthly minimum temperature; Tmin, the monthly minimum temperature; Prec, mean monthly precipitation. *p < 0.05; **p < 0.01; ***p < 0.001.

In terms of functional guilds (Table 3), ectomycorrhizal fungi were significantly negatively correlated with climate factors (*p* < 0.001), while endophyte and undefined saprotroph were significantly positively correlated with climate factors (*p* < 0.001). In addition, pH was negatively correlated with the relative abundance of endophyte and undefined saprotroph (*p* < 0.01). Moreover, plant pathogen was significantly negatively correlated with SOC (*p* < 0.05) and ericoid mycorrhizal fungi was significantly negatively correlated with TN (*p* < 0.05).

### 3.6. Environmental drivers of fungal community structure

To evaluate the relationship of fungal community structure with environmental variables at spatial scales, redundancy analysis (RDA) of the fungal community and the Mantel test were implemented.

**Figure 6:**
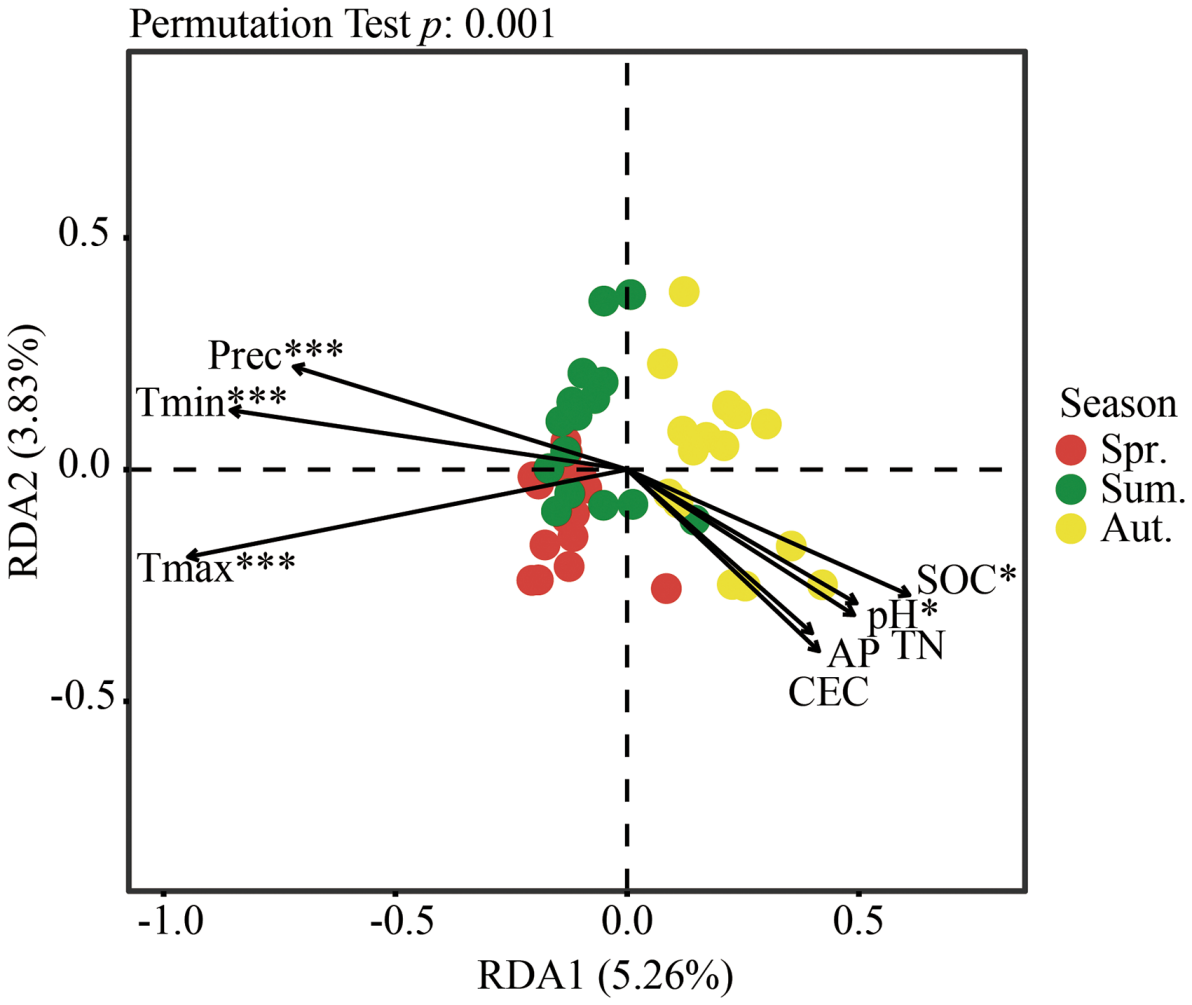
Redundancy analysis (RDA) of fungal communities at the OTU level and environmental variables. The envfit function was used to test the significance of each physicochemical factor in RDA. *p < 0.05; ***p < 0.001.

The RDA result showed that the first two axes explained 9.09% of the total variation in fungi (Figure 6). Climate factors had significant effects on community structure, while only SOC and pH in soil physicochemical properties had significant effects on community structure between seasons.

Based on the Mantel test (Table 4, *p* < 0.05), the climate factor was remarkably correlated with the fungal community structure. The main factors that affected the fungal community of the soil were TN, SOC, and CEC.

**Table 4:** Correlations between fungal community structure and environmental variables assessed by Mantel tests.

Variable	TN	SOC	pH	CEC	AP	Tmax	Tmin	Prec	Total
*R*	0.164	0.178	−0.043	0.122	−0.076	0.184	0.101	0.101	0.176
*p*	0.023	0.019	0.748	0.029	0.815	0.001	0.023	0.026	0.007

SOC, soil organic carbon; AP, available phosphorus; pH, pH value; CEC, cation exchange capacity; Tmax, the monthly minimum temperature; Tmin, the monthly minimum temperature; Prec, mean monthly precipitation.

In summary, whether a factor was considered separately or all environmental variables were considered, temperature and precipitation were extremely significant factors affecting the community structure, and except for AP, soil physicochemical properties had more or less influence on the community structure.

## 4. Discussion

### 4.1. Seasonal variation in soil properties and climatic factors

Temperature and precipitation are the most significant changes brought about by seasons. According to the existing research, the cold temperate zone where the Aershan National Forest Park is located in long and cold in winter (Li et al., 2011). The growing season of plants is short, and seasonal changes have a profound impact on the growth cycle of plants, which also mobilize the structural changes of fungi in the underground soil (Zhang and Fu, 2021). In this study, the temperature and precipitation increased first and then decreased, and the temperature and precipitation in autumn were much lower than those in spring, even though the lowest temperature was below zero.

In the soil physicochemical properties, TN and AP have identical change trends as the climate, while pH first decreases and then increases. SOC showed a continuous increase with the season, while CEC continued to decline with the season. Worldwide, the storage of SOC usually increases with the decrease in annual average temperature, and the carbon element in cold regions is more enriched (Stockmann et al., 2013), which is consistent with the highest content of SOC in winter in this study. Soil CEC could affect the growth and development of plants by significantly changing leaf Ca and Mg absorption, chlorophyll content, and catalase activity (Cui et al., 2021). In winter, the demand of plants for CEC decreases, which cannot drive the accumulation of CEC in the soil and may cause the loss of CEC in the soil.

### 4.2. The response of fungal communities to seasonal variation

Recent studies have shown that there are many factors that affect the diversity of soil fungi. Xie and Yin (2022) showed that soil fungal diversity and richness in broad-leaved forests were higher than those in conifer forests. Fungal community diversity and composition are significantly driven by soil pH, available nitrogen, available phosphorus, moisture, organic carbon, fine root biomass and root tissue density (Beauregard et al., 2010; Zhou et al., 2016b; Tauro et al., 2021). Soil pH is the dominant driver that is significantly related to fungal alpha diversity (Wang et al., 2015; Adamo et al., 2021). Seasonal climate change directly affects the diversity of soil fungi, and indirectly affects it by affecting soil properties and root variables (Xie and Yin, 2022). In neutral pH soil, soil quality decreased along the altitudinal gradient, indicating that microbial diversity was likely constrained by climatic conditions (Bayranvand et al., 2021). Climatic factors, followed by edaphic and spatial patterning, are the best predictors of soil community composition at the global scale (Tedersoo et al., 2014). In our study, the diversity of the fungal community changed with the seasons. The diversity of fungi in summer was significantly higher than that in spring and winter, which was consistent with the seasonal change in the diversity of fungi in subtropical orchard soil (Koide et al., 2005). Climate factors and soil pH significantly affect the diversity of soil fungi in *L. gmelinii* forests.

According to Lauber et al. (2008) the composition of the fungal community is most closely related to the change in soil nutrients. There was a significant correlation between the variety in fungal community composition and the availability of soil carbon and nitrogen (Zhang et al., 2016; Sanka Loganathachetti et al., 2017). We analyzed the correlation between dominant fungal genera and soil properties and climate factors, and the results showed that *Inocybe* and *Mortierella* among the top five dominant genera of relative abundance had a very close relationship with climate factors and pH, while *Sebacina* was significantly positively correlated with TN, SOC and CEC in soil physicochemical properties. However, in our previous study on the soil microbial community of *L. gmelinii*, the influence of soil physicochemical properties on the composition of the fungal community was not significant (Zhao et al., 2022). We thought that the difference in soil physicochemical properties in the same season was not enough to cause the difference in soil fungal diversity.

The dominant soil fungal communities could adapt and respond to climate change by altering the proportion of different dominant fungal groups by responding to moisture patterns (Huang et al., 2021). Tedersoo et al. (2014) found that climatic factors, followed by edaphic and spatial patterning, were the best predictors of soil community composition at the global scale. Variations in soil fungal community composition across seasons were attributed to their functional adaptation. Previously, soil fungi in northern coniferous forests were dominated by saprophytic fungi at the end of winter and were gradually replaced by ectomycorrhizal fungi in the growing season (Santalahti et al., 2016). The relative abundance of ectomycorrhizal fungi showed absolute dominance in all 3 months, and increased when the temperature decreased. Zeng et al. (2022) presented that ectomycorrhizal taxa dominate functional guild in subtropical evergreen broad-leaved forests. Ectomycorrhizal fungi are affected by multiple factors, and temperature and precipitation had a significant effect on them in our study. In the study of Yang T. et al. (2020), the observed decline in ectomycorrhizal fungal richness may be related to the fire-induced mortality of ectomycorrhizal plant hosts. Accordingly, we suppose that the seasonal dynamics of plants also influence the changes in the relative abundance of ectomycorrhizal fungi, except for climatic factors in this study.

Microbial community structures were strongly affected by seasonal variations. In the study of microbial community structure at different elevations, bacterial and fungal community structures exhibited a pronounced annual cycle (Lazzaro et al., 2015; Turatsinze et al., 2021). Fungal communities differed among seasons, equivalent to the community turnover observed over thousands of kilometers in space. Climate covariates explained some spatial–temporal effects (Averill et al., 2019). According to the results of the RDA and Mantel test in this study, inter-seasonal climate factors play a core role in fungal community structure. In addition, Adamo et al. (2021) showed that soil parameters were the most important driving forces shaping soil fungal communities at the regional scale in the Mediterranean pine forests. The species richness and diversity of ectomycorrhizal fungi declined following long-term nitrogen addition in a temperate forest, while ascomycetes and saprotrophs responded positively to N enrichment (Morrison et al., 2016). SOC may affect the structure of the soil fungal community (Sul et al., 2013; Li et al., 2017), because the abundance of cellulose-degrading fungi may be linked to SOC fractions and may respond to changes in SOC fractions differently. Soil available phosphorus and nitrogen are influential factors shaping fungal communities (Tauro et al., 2021). In a previous study, the fungal community was less strongly affected by pH, because fungi generally exhibit wide pH ranges for optimal growth (Rousk et al., 2010; Wang et al., 2015). In this study, combined with RDA and Mantel test, TN, SOC, pH, CEC in soil properties and climate factors influence the structure of the soil fungal community, among which climate factors have a pivotal role.

## 5. Conclusion

In this study, we demonstrated that season had a strong effect on fungal community structures. This is mainly due to the impact of seasonal climate change on soil fungi. Soil properties will also cause changes in the diversity and composition of fungal communities. However, we believe that climate factors have a more significant influence on the structure of soil fungal communities than soil properties. In addition, we found that ectomycorrhizal fungi dominated the functional guilds in the *L. gmelinii* forest.

## Data availability statement

The datasets presented in this study can be found in online repositories. The names of the repository/repositories and accession number(s) can be found in the article/Supplementary material.

## Author contributions

B-KC designed the research. B-KC, WZ, D-DW, K-CH, SL, and MR prepared the samples. WZ conducted the molecular experiments and analyzed the data. WZ, Y-FS, J-NL, and B-KC drafted the manuscript. All authors have read and agreed to the published version of the manuscript.

## Funding

The research is supported by the National Natural Science Foundation of China (nos. 32270010, U2003211, and 31870008) and Beijing Forestry University Outstanding Young Talent Cultivation Project (no. 2019JQ03016).

## Conflict of interest

The authors declare that the research was conducted in the absence of any commercial or financial relationships that could be construed as a potential conflict of interest.

## Publisher’s note

All claims expressed in this article are solely those of the authors and do not necessarily represent those of their affiliated organizations, or those of the publisher, the editors and the reviewers. Any product that may be evaluated in this article, or claim that may be made by its manufacturer, is not guaranteed or endorsed by the publisher.

## Footnotes

^1^ https://www.worldclim.org/data/monthlywth.html#
